# Inadvertent hypoventilation during pressure-controlled ventilation with volume guarantee mode of Aisys® anesthesia machine

**DOI:** 10.1186/s40981-020-00341-8

**Published:** 2020-05-11

**Authors:** Mai Nabatame, Shuya Kiyama, Shoichi Uezono

**Affiliations:** Department of Anesthesiology, School of Medicine, Jikei University, Nishi-Shimbashi 3-25-8, Minato-ku, Tokyo, 105-8462 Japan

To the Editor:

During pressure-controlled ventilation with volume guarantee (PCV-VG) of Aisys® anesthesia Carestation (GE Healthcare, Helsinki, Finland), peak inspiratory pressure is automatically adjusted to deliver set tidal volume. The anesthesia machine measures tidal volume by integrating flow. We wish to report inadvertent hypoventilation because tidal volume was not measured correctly, which led to erroneous adjustment of peak inspiratory pressure.

During craniotomy for a brain tumor, anesthesia had been maintained by low-flow desflurane (fresh gas flow 0.5 l per minute). A hydrophobic filter had not been placed in the respiratory circuit. For intraoperative ventilation, PCV-VG mode was set to deliver tidal volume of 500 mL, approximately 7 ml per patient’s body weight. In the initial three and a half hours, ventilation status was stable with measured tidal volume of 490 ml, peak inspiratory pressure was 19 cmH_2_O, and partial pressure of end-tidal carbon dioxide (ETCO_2_) was between 30 and 35 mmHg. Then, ETCO_2_ gradually increased towards 50 mmHg in approximately 15 min. At this time, tidal volume was 800 ml and peak inspiratory pressure had been automatically reduced to 12 cmH_2_O. PCV-VG mode was turned off, and patient’s lungs were ventilated manually. Measured tidal volume was 1000 ml when the lungs were inflated to 19 cmH_2_O. We suspected that value of tidal volume shown on the monitor may be incorrect. A respiratory assembly which includes inspiratory and expiratory flow sensors was replaced. After replacement of this assembly, tidal volume and peak airway pressure returned to initial values (600 ml and 19 cmH_2_O) within 10 min of recognition of the problem. ETCO_2_ gradually decreased to 34 mmHg, and subsequent perioperative course was uneventful. A clinical engineer noticed that there was water condensation in the corrugated tubing of the respiratory circuit.

Postoperatively, we monitored tidal volume during PCV-VG using a test lung and an in-circuit humidifier with support of the manufacturer engineers. However, the similar phenomenon as above could not be reproduced. Therefore, we deliberately added a few milliliters of water to the flow sensor, and suddenly, a much greater tidal volume was shown on the anesthesia machine display.

Wax and Neustein [1] have reported erroneous display of expired tidal volume while using the Aisys® anesthesia machine, but in their case, tidal volume was less than that actually delivered. We could not find any other reports in which tidal volume was greater than the actual value. On what condition a faulty flow sensor leads to either larger or smaller tidal volume warrants further study.

During prolonged low-flow inhalation anesthesia, substantial amount of water can accumulate in a respiratory circuit. Water condensation in the flow sensor may cause erroneous measurement of tidal volume. In PCV-VG mode, peak inspiratory pressure may then be adjusted inappropriately and leads to inadvertent hypercapnia or hypocapnia, depending on miscalculated tidal volume. We wish to advise readers that excess water be drained regularly to prevent malfunction of PCV-VG mode. When tidal volume is suspected to be incorrect, an analog device such as a Wright spirometer can be used to measure tidal volume.

## Data Availability

Not applicable.
